# The biodiversity hypothesis and allergic disease: world allergy organization position statement

**DOI:** 10.1186/1939-4551-6-3

**Published:** 2013-01-31

**Authors:** Tari Haahtela, Stephen Holgate, Ruby Pawankar, Cezmi A Akdis, Suwat Benjaponpitak, Luis Caraballo, Jeffrey Demain, Jay Portnoy, Leena von Hertzen

**Affiliations:** 1 Skin and Allergy Hospital, Helsinki University Hospital, PO Box 160, 00029, Helsinki, HUCH, Finland; 2 School of Medicine, University of Southampton, Southampton, UK; 3 Nippon Medical School, Tokyo, Japan; 4 Swiss Institute of Allergy and Asthma Research, University of Zurich, Davos, Switzerland; 5 Department of Pediatrics, Ramathibodi Hospital, Mahidol University, Bangkok, Thailand; 6 Institute for Immunological Research, University of Cartagena, Cartagena, Colombia; 7 Allergy, Asthma & Immunology Center of Alaska, Dept of Pediatrics, University of Washington, Washington, USA; 8 University of Missouri-Kansas City School of Medicine, Missouri, USA

**Keywords:** Allergy plan, Biodiversity, Civilization disease, Epigenetics, Immune dysfunction, Microbiota, Microbiome, Urbanization

## Abstract

Biodiversity loss and climate change secondary to human activities are now being associated with various adverse health effects. However, less attention is being paid to the effects of biodiversity loss on environmental and commensal (indigenous) microbiotas. Metagenomic and other studies of healthy and diseased individuals reveal that reduced biodiversity and alterations in the composition of the gut and skin microbiota are associated with various inflammatory conditions, including asthma, allergic and inflammatory bowel diseases (IBD), type1 diabetes, and obesity. Altered indigenous microbiota and the general microbial deprivation characterizing the lifestyle of urban people in affluent countries appear to be risk factors for immune dysregulation and impaired tolerance. The risk is further enhanced by physical inactivity and a western diet poor in fresh fruit and vegetables, which may act in synergy with dysbiosis of the gut flora. Studies of immigrants moving from non-affluent to affluent regions indicate that tolerance mechanisms can rapidly become impaired in microbe-poor environments. The data on microbial deprivation and immune dysfunction as they relate to biodiversity loss are evaluated in this Statement of World Allergy Organization (WAO). We propose that biodiversity, the variability among living organisms from all sources are closely related, at both the macro- and micro-levels. Loss of the macrodiversity is associated with shrinking of the microdiversity, which is associated with alterations of the indigenous microbiota. Data on behavioural means to induce tolerance are outlined and a proposal made for a Global Allergy Plan to prevent and reduce the global allergy burden for affected individuals and the societies in which they live.

## Introduction

**Figure 1:**
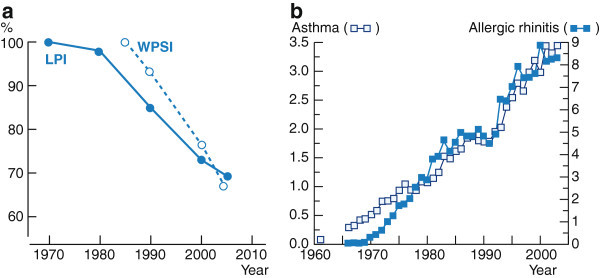
**Two global megatrends in biodiversity and public health.** (**a**) Declining biodiversity (percentage change) since 1970 as measured by two indices. WPSI=Waterbird Population Status Index; LPI=Living Planet Index [14]. (**b**) Increasing trends in the prevalence of inflammatory civilization diseases, asthma and allergic rhinitis among military conscripts in 1966-2003 [165] as an example (modified from ref. [14]).

Biodiversity loss is a global concern with a variety of possible adverse consequences for humanity. The reasons for this loss are complex and are in large part due to the consequence of industrialization, pollution and utilization of chemicals, which impact the environment and the microorganisms with which humans have lived since time immemorial. In recent years, attention has been paid to the potential health effects of this altered biosphere. Indeed, the two global megatrends, one in the state of biodiversity and the other in the prevalence of mucosal inflammatory diseases, may be more closely linked than is commonly recognized (Figure 1).

Inflammation is a cardinal feature of asthma and allergic diseases, autoimmune diseases, and many forms of cancer [1], but more recently less tangible associations have been linked to these trends such as an increased incidence of depression associated with inflammatory markers [2]. Thus far, the increase in the prevalence of inflammatory disorders is a phenomenon largely restricted to the developed world, while such disorders are still uncommon among populations in non-affluent regions, i.e. those regions, which still have more traditional non-urban lifestyles [3,4]. Adoption of cultural patterns and nutritional habits from affluent countries, together with declining frequency and severity of intestinal parasitic infections, are rapidly changing the allergy trends upwards also in many developing countries [5]. Not unexpectedly, loss of traditional cultures and loss of biodiversity are likely linked and as such, driven by shared factors such as increased urbanization, commercialization and widespread use of chemicals [6].

Previous studies reveal that microbe-rich environments confer protection against allergic and autoimmune diseases [3,4,7], but it is likely that declining biodiversity is more generally responsible for human immune dysfunction. Hanski et al. [8] showed recently, that compared with healthy adolescents the atopic individuals had lower environmental biodiversity **–** in the form of species richness of native flowering plants and in the land use type **–** in the surroundings of their homes. The atopic adolescents also had significantly lower generic diversity of Gram-negative gammaproteobacteria on their skin. Furthermore, the abundance of the genus *Acinetobacteria* on the skin was positively correlated with the peripheral mononuclear cell expression of IL-10, a key anti-inflammatory cytokine in immunological tolerance. Gammaproteobacteria are common in the soil, but are particularly dominant in above-ground vegetation, such as flowering plants.

The line of evidence that the declining biodiversity of plants, animals, and their natural habitats is associated with changes in the interactions between humans and microbes, and that these interactions might be causally related to the increasing prevalence of asthma and allergy as well as other inflammatory disorders, is reviewed in this paper.

### Focus on environmental factors

Irrespective of major efforts to clarify the genetic causes, which predispose to the onset of asthma and allergic diseases, the results remain rather modest [9], underscoring the genetic complexity of these multi-trait diseases. Increased attention is now focused on critical environmental factors in the search for the origins of these diseases. Studies of immigrants, epigenetic studies and mapping of the gut microbiota have provided compelling evidence that the environment can fundamentally modulate immune function in humans (Table 1). Poorly developed or broken immune tolerance plays a role in the pathogenesis of many diseases such as allergy and asthma, autoimmunity, cancer, chronic infections, abortions [10].

**Table 1:** Evidence of the immunomodulatory capacity of the living environment in humans

**Evidence from**	**Altered susceptibility to**	**References**
**Immigrant studies**	Allergic diseases	[74,75,77,78]
	Type 1 diabetes	[73]
	Multiple sclerosis	[76]
	Obesity, type 2 diabetes	[79]
	Depression	[80]
	Cancers	[81]
**Epigenetic studies**		
Microbial deprivation	Allergic diseases	[111,117,118]
Air pollution	Asthma deterioration	[119]
**Studies on the gut and skin microbiota**		
Microbial deprivation, altered composition	Allergic diseases	[8,51-54]

Data on altered susceptibility to inflammatory diseases are for immigrants moving from less affluent to affluent countries, except in ref [76].

Living in urban environments with higher exposure to chemicals and with reduced green space with the consequence of limited plant, animal and microbial life is associated with immune dysfunction and impaired tolerance in humans [11]. Reduced contact with nature and environmental microbiota appears to be linked with a range of civilization diseases, including allergy and type 1 diabetes [12]. Of considerable concern is that these chronic inflammatory diseases are becoming increasingly prevalent in low and middle income countries in parallel to their improving economic development and adoption of western-type urbanization. It is possible that a more biodiverse exposure confers more protection, not only against infectious diseases [13], but also against chronic inflammatory (and possibly malignant) diseases.

Novel methodologies now allow accurate determination of microbial communities in various ecosystems. Despite this advance in technology, hardly anything is known about the interactions between environmental and indigenous microbiotas. How are reduced biodiversity and the change in the micro-organism profile found in urban and more affluent environments reflected in the indigenous human microbiotas? What are the effects of biodiversity loss of plants and animals on environmental microbiota?

We propose that biodiversity at the level of macrobiota and microbiota are interrelated in that biodiversity loss of the former is likely to be associated with loss of diversity of the latter [14]. Moreover, biodiversity loss leads to reduced interaction between environmental and human microbiotas. This in turn may lead to immune dysfunction and impaired tolerance mechanisms in humans.

### Concepts of biodiversity

By definition, biodiversity is ‘the variability among living organisms from all sources, including, *inter alia,* terrestrial, marine and other aquatic ecosystems and the ecological complexes of which they are part. This includes diversity within species, between species and of ecosystems’ [15]. In practice, the key elements of biodiversity include genetic diversity of populations and species; the richness of local and global species; the spatial extent and the state of natural habitats; and the functioning of ecosystems that provide various essential services to mankind.

The year 2010, the United Nations’ International Year of Biodiversity, was to be the turning point for biodiversity decline, but a recent comprehensive report shows that this target has not yet been met; the rate of biodiversity loss does not show signs of decreasing, and worryingly, the indicators which reflect the various pressures on biodiversity continue to increase [16]. For example, one-third of the sufficiently well-defined species of animals and plants are currently classified as threatened (56000 species) [17].

Although the Convention of Biological Diversity [15] is primarily concerned with plants and animals, biodiversity also includes micro-organisms, which are less visible but comprise the bulk of living matter on our Earth [18]. Biodiversity, according to the definition, concerns both environmental and commensal microbiota. Thus far, little attention has been paid to the biodiversity of environmental microbiota.

### Biodiversity and human health

There is a rich literature to demonstrate that natural environments are of vital importance to the physical and mental health of humans [19], and as a consequence, biodiversity should be integrated into the conventional measures of well-being and recognized as a global public good requiring conscious collective choices [20].

A recent large study found that the annual prevalence rate of most disease clusters was lower in environments where people were living with more green space (10% or more than the average) within a 1 km radius. This effect was most pronounced for depression and anxiety, but also significant for asthma/COPD, diabetes and coronary heart disease [21]. Green space, including forests and natural areas as well as parks, may act as a buffer between stressful life-events and health [22]. Aligned to this concept, better self-reported health and lower stress scores also have been reported to be inversely associated with the distance to green space [23].

A meta-analysis of 10 intervention studies reveal that ‘green exercise’ (activities in natural environments), even of short duration, significantly improved the physical and mental health [24]. Of importance, physical activity in a natural environment versus other settings, appears to have more beneficial health effects [25].

Several on-going studies have examined the health effects of forests [26]. Compared with urban settings, forest environments are associated with lower cortisol level, lower pulse rate and blood pressure as well as with higher parasympathetic and lower sympathetic nerve activity in healthy individuals [27]. It is increasingly recognized that exposure to nature, and forest environments in particular has the potential to substantially improve human health [26].

The health effects of natural environments are obvious, but difficult to examine experimentally. Accumulating evidence underlines the critical role for micro-organisms in the normal development and maintenance of mucosal integrity and tolerance [28,29]. Urbanization and general loss of biodiversity, combined with sedentary indoor lifestyles have been a principle driving factor leading to microbial deprivation [14,30] and may play a significant role in the epidemics of inflammatory diseases. Nevertheless, more data and better understanding of the relevant mechanisms underlying the ‘nature effect’ at the cellular and molecular levels are necessary.

### Immune regulation – mechanisms of tissue integrity and homeostasis

Various immune cells must interact with microbes for normal development and function. Protective mechanisms against inflammatory diseases involve the activation of the innate and regulatory networks by continuous exposure to microbial components via the skin, gut and respiratory tract. Humans have evolved with these micro-organisms, which do not elicit defensive immune responses, but rather induce immune regulatory circuits. A suddenly reduced abundance or diversity of these micro-organisms, previously ubiquitous, may have led to failures to regulate and restore appropriate immune and inflammatory responses [11].

Signaling via Toll-like receptors (TLRs) and other conserved pattern-recognizing molecules [31,32] that are present on/in various cells play a decisive role, not only in host defense against pathogens, but also to maintain epithelial cell homeostasis and tissue repair [28]. This apparently universal phenomenon has been shown in various tissues and occurs in wound healing [33]. Evidence from mice highlights the role of TLR stimulation to confer protection against inflammatory conditions, supporting the epidemiological studies of human populations living in a microbe-rich environment. The effects were mediated by induction of regulatory circuits [34,35] and by stimulating innate immune mechanisms in epithelial cells [36].

### Alterations in human microbiota – implications for immune dysregulation and inflammatory disorders

A fundamental role for micro-organisms in human health, whether indigenous or environmental, is becoming increasingly evident. Commensals are no longer considered as passive bystanders or transient passengers, but increasingly as active and essential participants in the development and maintenance of barrier function and immunological tolerance [37]. They are also involved in the programming of many aspects of T cell differentiation in co-operation with the host genome [29]. Mounting evidence also shows that alterations in the indigenous microbiota correlate with inflammatory disease states [38]. The indigenous flora may not only comprise bacteria and fungi, but also viruses and microscopic protozoans, although hardly any data on the latter are available.

This paper focuses on the gut and skin bacterial flora, the two microbiotas that have been studied in greatest detail. Although the current literature is dominated by data about the gut flora to maintain immune tolerance and health, the on-going metagenomic studies may demonstrate a significant role for microbiotas at other sites, including the respiratory tract [39] and skin [40].

#### Gut microbiota and inflammatory diseases

The human gut flora is thought to be an essential ‘organ’, which not only provides nutrients, but also regulates epithelial development and orchestrates innate immunity [41]. The gut community is dominated by only two phyla, *Firmicutes* and *Bacteroidetes,* and to a lesser extent by *Acinobacteria* and *Proteobacteria*, even though the populations of bacteria in the gut are huge and diverse at both the genus and species levels [41-43]. Recent metagenomic data indicate that any one individual harbours an assemblage of at least 160 species, which are partly shared with other individuals. The total number of bacterial species identified in a sample of 124 Europeans was 1000 to 1150 [44], suggesting a rather distinct composition of flora present in each individual.

The microbiota is variable and shows temporal fluctuation in early life. When a steady state is reached, the composition of the gut flora remains relatively stable over time, providing that no major changes in lifestyle or environment occur [45]. Early environmental exposures are thus considered the key determinant of adult gut microbiota [46], as is the type of diet consumed [47-50]. Clues about the influence of environmental factors on the development of the gut microbiota have been obtained from studies of infants in Estonia and Sweden and provide some of the first evidence that the composition of gut microflora between non-western and western children in early life differs and that these disparities could be associated with the manifestation of allergic diseases in later life [51-53]. Using novel molecular-based methods, a Swedish study revealed that a more diverse gut microbiota early in life is associated with protection against allergy at the age of 5 years [54].

**Figure 2:**
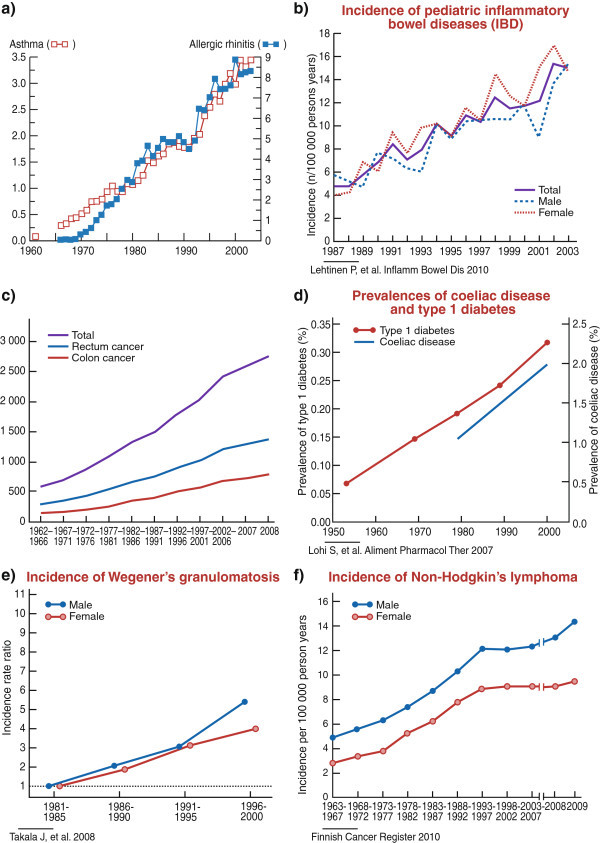
**Trends in a) prevalence of asthma and allergic rhinitis **[165]**, b) incidence of pediatric inflammatory bowel disease **[59]**, c) incidence of colon and rectum cancer **[168]**, and d) prevalence of coeliac disease and type 1 diabetes **[166]**, e) incidence of Wegener’s granulomatosis **[167]**, and f) incidence of non-Hodgkin’s lymphoma in Finland in 1950-2009 **[168]**.**

Dysbiosis, the reduced diversity and disturbed composition of the gut microbial community, not only has an influence on the occurrence of asthma and allergies, but also on other chronic and relapsing inflammatory conditions that include type 1 diabetes [55], inflammatory bowel disease (IBD) [56,57], obesity [42], and even psychiatric disorders, such as depression [58]. Studies in both mice and humans indicate that some common members of the normal microbiota could exert a special role in maintaining homeostasis and immune health [29,45,56,57]. A decrease or absence of these microbes in the colon has been shown to lead to impaired development of regulatory T lymphocytes (T reg cells), the T cell subset that mediates suppression of T-cell mediated inflammatory responses. Moreover, an imbalance of ‘pro-inflammatory’ and ‘anti-inflammatory’ microbes may also result in an increased susceptibility of the host to inflammatory diseases [29] and could explain the increase in paediatric inflammatory bowel disease (IBD) [59], one of the many inflammatory diseases being reported with increasing frequency in westernized countries (Figure 2).

Although it is unclear what is the cause and effect, the rich literature on the mechanisms for the development of epithelial cell tolerance and homeostasis referred to above and the experimental models using transfer of gut microbiota [55,60] support the concept of alternative stable states. Altered environmental microbiota and reduced signaling of TLR and other receptors cause immune dysfunction, enhancing the colonization and growth of a biased microbiota, thus creating a self-perpetuating circle to push the host-microbe interaction toward an ‘unhealthy’ state [14]. Collectively, sedentary lifestyle in affluent urban environments does not provide adequate microbial exposure to develop ‘healthy’ microbiota, an essential part of the immune system closely linked to the development and maintenance of epithelial cell tolerance and homeostasis. An indirect illustration of this concept is that faecal microbiota transplant has been successfully used to restore the microbiota balance in severe *Clostridium difficile* infections [61].

#### Skin microbiota

Skin flora, similarly to gut flora, can be viewed as a community or an organ composed of various cell lines that communicate with each other and with host cells. The use of global metagenomic approaches reveals that the microbial diversity of the skin is comparable to that in the gut [44,62], giving credence to the fact that the skin flora is a part of the immune system and able to shape the responses. In genetically predisposed individuals, various environmental factors may lead to inflammatory skin responses and impaired barrier function as seen with chronic eczema [63]. A good example is the possible role of staphylococci in predisposing to atopic eczema [40], but on a larger scale, the role of skin microbiota in barrier function is not yet fully elucidated.

Several metagenomic studies [62,64-66] demonstrate that the skin microbiota is composed mainly of members of the same four phyla that comprise the gut microbiota, although with dissimilar relative abundances [64-66]. An exciting and highly relevant study shows that each of these two body sites harbour its own characteristic microbiota, with little temporal variation [64,65]. The factors that determine these complex communities and their relationship to the host both to maintain health and predispose to disease open up a whole new field of inquiry into human homeostasis and its potential link to the wider environment.

As in the gastrointestinal tract, the skin microbial community includes both transient and resident microbiota [66,67], suggesting that at least a part of the skin flora is in a dynamic interaction with the environment. To probe these interactions, long-term studies are needed over lifetime, on the intensity and composition of the skin flora in people living in contrasting environments in different countries and socioeconomic conditions and their associations with health and disease.

#### Effect of antibiotics

Antibiotics modulate microbiota, and the effect may be long-term. One-week course affected the gut microbiota even for three years [68]. Recent systematic review by Murk et al. [69] indicated a slight increase in the risk of asthma, if antibiotic were used during pregnancy or in the first year of life. Hill et al. [70] showed how in antibiotic treated mice commensal-derived signals influenced basophil hematopoiesis and susceptibility to TH 2 cytokine-dependent inflammation and allergic disease. Cho et al. [71] increased adiposity in young mice by administering sub-therapeutic doses of antibiotics. Changes were observed in the gut microbiota as well as in copies of key genes involved in the metabolism of carbohydrates to short-chain fatty acids.

Altogether, antibiotic manipulation seems to alter, especially in early-life, metabolic homeostasis and increase risk of allergic and other inflammatory conditions.

### Immigration reshaping immune regulation

Immigrant studies have provided invaluable evidence for the immunoregulatory capacity of the living environment. When immigrants move from areas of low to high prevalence of disease, they have a good health status at the time of arrival (healthy immigrant effect), but this declines thereafter and converges to that of the natives [72] or even becomes worse. The immunomodulatory changes appear to occur within 10 years after arrival [72-75], and these changes are not restricted to young people only but also occur in adults as well [76,77]. This immunomodulation by “cultural adaptation”, which leads to changes in disease susceptibility, appears to be universal and has been reported for various inflammatory diseases, including asthma and allergic diseases [74,75,77,78], autoimmune diseases, such as type 1 diabetes [73] and multiple sclerosis [76], obesity and type 2 diabetes [79], depression [80] and civilization cancers [81].

Of note, this list of disorders is almost identical to the disease spectrum associated with altered gut microbiota as discussed above. Recently-arrived individuals may experience a particularly dramatic reduction in health status, even if they are younger than the native residents and earlier immigrants. The relatively rapid change in health status is best explained by the dietary factors affecting gut microbiota. In addition to dietary factors and environmental exposures, psychological factors, such as loss of socioeconomic status contributing to stress and depression [72], are likely to play a key role in the immune modulation observed in immigrants.

### Additional risk factors

#### Air pollution

Indoor and outdoor pollution is a major environmental risk factor for asthma and allergy not only increasing the prevalences of long-term symptoms but also acute attacks [82]. The association studies indicate that ambient air pollution is connected to asthma, rhinitis, rhinoconjunctivitis, acute respiratory infections, the need for asthma drugs, and hospital admissions because of respiratory symptoms. Indoor tobacco smoke is the most obvious risk factor, which can be reduced by legislation and education. In Turkey a tobacco ban initiated in 2009 resulted in a 24% decrease in respiratory emergency visits in Istanbul in one year [83].

The main ambient air pollutants are derived from fuel combustion (traffic and various industrial sources like power plants and refineries). Fine particulate matter, nitrogen and sulphur compounds (NOx, SOx) are the most important pollutants emitted directly to the atmosphere. Ozone (O3) is produced by the reaction of sunlight with air containing hydrocarbons and NOx. In China, ambient air pollution is associated with 300 000 deaths and 20 million cases of respiratory illnesses annually [84].

The most important sources of indoor air pollutants, in addition to tobacco smoke are biomass fuels, like wood and coal, for heating and cooking, cleaning and washing products and mold/dampness. Conservative estimates indicate that indoor air pollution may be responsible for nearly two million deaths per year in developing countries [85].

#### Climate change

The Intergovernmental Panel on Climate Change (IPCC) defines climate change as a “change in the state of the climate that can be identified (e.g. using statistical tests) by changes in the mean and/or the variability of its properties and that persists for an extended period, typically decades or longer. It refers to any change in climate over time, whether due to natural variability or as a result of human activity.” The IPCC further states that warming of our climate system is unequivocal, demonstrated by increases in global air and ocean temperatures in addition to widespread melting of snow and ice as well as rising sea levels [86]. Climate change is resulting in chemical, physical and biologic stressors that have far reaching consequences to health [87].

The prevalence of asthma and allergic rhinitis has markedly increased globally since the 1960s [88,89]. Pollen is an important trigger in many patients with allergies and asthma. Air pollution and higher concentrations of CO2-induced increases in levels and allergenicity of allergenic pollens may be contributing to increasing prevalence of allergic disease and asthma, with climate change a plausible explanation for both [90].

Climate change has direct impacts on aeroallergens, in particular pollens and mold spores and allergic diseases [90-92]. Pre-Industrial CO2 levels in 1870 were 280 ppm, followed by a steady increase of 35% by 2005 to 379 ppm, with urban areas exhibiting the highest levels [87]. Several studies have demonstrated direct correlations between rising CO2 and increases in both pollen and biomass levels, as well as increased allergenicity of the pollen [93,94]. Ziska, et al. [94] tested the hypothesis that ragweed pollen levels were impacted by pre-industrial revolution CO2 level (280 ppm), current CO2 levels (370 ppm) and CO2 levels (600 ppm), projected level for the year 2100. All variables remained constant using a greenhouse environment, with the exception of rising CO2 levels. There was a 132% increase in ragweed pollen from pre-industrial to current and an additional 90% increase in pollen level at projected 2100 levels. In the greater Baltimore area between 2000 and 2001, Ziska demonstrated that the urban levels of CO2 were 30% higher and temperature 2 degrees Celsius higher than surrounding rural areas. A fundamental aspect of climate change is the potential shift in flowering phenology and pollen initiation associated with milder winters and warmer seasonal air temperature. Earlier floral anthesis has been suggested, in turn, to have a role in human disease by increasing the time of exposure to pollen that cause allergic rhinitis and related asthma. In the urban area, the ragweed plants produced 189% more pollen, compared with the surrounding rural area [95].

Correlation between earlier ragweed pollen start dates, prolonged pollination cycles and increasing latitudes of ragweed growth in North America has also been reported, with a nearly linear increase from Oklahoma City with 1 day earlier from 1995 to 2009 to a high of 27 days earlier in Saskatoon, Canada [96]. In addition to increases in pollen levels and prolonged pollination cycles, there is also evidence of an impact on pollen spatial distribution, dispersal of pollen and increased allergenicity of the pollens [97,98].

D´Amato et al. [99] have described the potential of climate change to alter atmospheric circulation patterns, which contribute to long distance transport of allergenic pollen. Extreme events like thunderstorms and cyclones have an impact on aeroallergens increasing the risk of sudden exposure.

In high CO2 environments, birch pollen exhibited increased bet-v-1 specific IgE binding compared to baseline levels, p<0.05 [100]. Interestingly, similar findings have been demonstrated with molds, specifically *Alternaria alternata*, with a significant increase in spore production as well as an increase in allergenicity in high CO2 environments compared to low [92]. These effects do not seem limited to pollen and spores. Increased toxicity (allergenicity) has also been demonstrated in poison ivy subjected to elevated atmospheric CO2 [101].

A recent retrospective study looked at 27 years of pollen counts compared with correlating prevalence of the development of allergen sensitivity. Significant increases in pollen levels and duration of the pollen season of cypress (+18 days), olive (+18 days) and *parietaria* (+85 days) were demonstrated, with no significant change in birch or grass. The percentage of patients sensitized to those pollens significantly increased (p<0.05), while there was no change in the prevalence of dust mite sensitivity (used as a control). The authors went on to look at climate variables, finding significant correlations with rising levels and prolonged season with increasing temperatures and increased solar irradiation [102].

Impact of climate change on indoor aeroallergens, such as molds, house dust mites, cockroaches, rodents, and others pests have received less attention than those on outdoor allergens. For example, increased relative humidity will increase the risk for mold growth and heavy periodic rainfalls stress the buildings to manage excess water flow [103].

### Gene – environment interaction

Disposition to atopy and associated allergic disorders are strongly heritable. In addition, most atopic diseases coexist in the same patients suggesting overlapping mechanisms that involve in large part Th2-related inflammatory mechanisms. Atopy that predisposes to enhanced sensitisation to common allergens is only one genetic factor driving the heritability of allergic disease, another are genes that predispose to expression of the atopic phenotype in a particular organ. A good example is atopic dermatitis (eczema). The mutations of the filaggrin gene may increase skin permeability and predispose to allergen penetration and sensitisation of the skin (also upper airways) and are closely associated with severe disease [104]. A breakdown of epithelial barrier function is also an emerging factor in other allergic diseases such as food allergy, rhinosinusitis and asthma although other defects beyond filaggrin are involved [105].

The large geographic variations in the prevalence of allergic diseases and rising trends reported in the low and middle income countries have been linked to some aspects of Western lifestyles, but what these factors are have so far eluded definition. The genetic changes in populations over the time when these trends have occurred cannot account for the rise in allergic disease associated with socioeconomic gradients. As discussed earlier, changes in microbial exposure, diet and exposure to pollutants and chemicals (not discussed further in the present paper) are all being pursued as possibilities. Most likely, it will be multiple environmental changes interacting at different time points across the life course (including pregnancy) that interact to predispose to allergic sensitisation and atopic disease.

Although there have been many examples of genetic mutations of pathogen recognition receptors (PRR), especially toll-like receptors influencing the association between atopic disease including asthma, genome- wide association studies (GWAS) have in this respect been disappointing [106].

This is especially the case in the protective influence of early life microbial exposure in farming and related environments and anthroposophic lifestyles, and novel genes associated with asthma and atopy. The exception is exposure to maternal and environmental tobacco smoke that has been reported to increase the strength of association between some novel genes and asthma e.g. the ORMDL3 locus on 17q21 and PCDH1 on 5q31-q33 [107]. What are now needed are GWAS in which environmental measures and individual responses are incorporated at the start in order to uncover those disease-related genes that are most sensitive to particular environmental exposures. Another way of seeking such genes is to explore epigenetic influences.

### Epigenetic modulation

Epigenetic changes, functionally relevant, environmentally induced genome modifications that can be heritable, but are unrelated to DNA sequence changes [108], has received much attention over the past few years as an explanation of immune modulation by environmental exposure. Environmental factors are key players in activating or silencing genes by altering DNA (CpG island) and chromatin histone acetylation, methylation and phosphorylation thereby altering chromatin structure, or through the inhibitory effects of microRNAs on gene translation, thus modifying disease susceptibility in individuals.

Epigenetic changes are likely to be of major importance in contributing to phenotypic expression of complex disease and are highly disease- and organ- specific. GWAS of several complex diseases have identified a number of genetic variants, but these variants only explain a minor part of disease susceptibility. For example, over 45 single nucleotide polymorphisms (SNP) associated with Type 2 diabetes account for only 10% of the phenotype. In the case of asthma, the EU GABRIEL collaboration reported highly significant genome-wide associations with loci on chromosomes-17 (*ORMDL3/GSDMB), -*2 (*IL1RL1/IL18R,* -6 (*HLA-DQ* gene cluster), -9 (*IL33), -*15 (*SMAD3)* and -22 (*IL2RB*) that is claimed to account for up to 50% of asthma risk across the life course [109]. Nevertheless, only a small proportion of the heritability of asthma can be accounted for by novel genes identified by GWAS. The missing heritability may in part result from non-synonymous variants with high penetrance as has been reported in some cases of extreme obesity.

The involvement of epigenetic changes in this ‘missing heritability’ [108] is also apparent, but still needs to be verified. Studies of disease discordance in monozygotic (MZ) twins indicate that significant epigenetic modulation occurs *in utero*. A study among newborn twins highlights the importance of the intrauterine period for establishing epigenetic variability in humans [110]. It is likely that many factors will influence epigenetic expression of specific genes beyond disease including, but not limited to, age, sex, shared environments, diet and smoking.

A microbe-rich environment has been shown to induce both pro-inflammatory and regulatory circuits very early in life [111], indicating early activation of the relevant genes. Prenatal exposure to high levels of microbial products e.g. milking parlours and cow sheds is especially protective. Such exposures lead to high circulating levels of Tregs, so that in mice, selective depletion of Tregs during the allergen sensitisation phase dramatically enhances experimental allergic lung inflammation [112]. One study in humans has shown that maternal cells have the capacity to traverse the placenta and enter fetal lymph nodes to induce Tregs with suppressive functions to prevent anti-maternal immunity [113]. Recently, reduced human placental Foxp3 has been shown to be associated with subsequent infant development of allergic disease [114].

Further support for maternal programming to protect against infant allergen sensitisation comes from exposing pregnant mice to the Gram-negative, non-pathogenic bacterium *Acinetobacter lwoffii* F78 found in cow sheds and which has been shown to activate TLR2, 3, 4, 7, and 9. Combining prenatal exposure to *A. lwoffii* F78 with a mouse model of Th2-mediated airway inflammation leads to protection from development of an allergic lung phenotype in the offspring [115,116]. The mechanism involves up-regulation of maternal lung TLR expression, initiation of a transient acute local Th1-like inflammatory response, systemic release of inflammatory cytokines and a down-regulation of TLR and cytokine expression in the placenta. As the offspring from *A. lwoffii* F78-treated TLR2/3/4/7/9^−/−^mothers were no longer protected from Th2-type airway responses to inhaled antigen, maternal TLR signalling appears crucial for mediating the transfer of this transgenerational protective effect. Most recently, the inhibitory effect of *A. lwoffii* F78 on the development of an allergic lung phenotype in the offspring has been shown to be IFN-γ -dependent and epigenetically controlled through chromatin histone 4 (H4) acetylation of the *IFNG* promoter of CD4^+^ T cells with inhibition of H4 acetylation abolishing the Th2-protective phenotype [117,118].

Traffic-related air pollution is an example of an environmental factor that may increase disease susceptibility due to epigenetic changes. A study among school children with and without asthma living in areas with contrasting levels of ambient air pollution showed that pollution is associated with increased methylation of CpG islands in the Foxp3 locus, reduced T reg cell function and increased asthma severity scores. A dose-dependent relationship between the exposure to air pollution and Treg cell function and/or Foxp3 methylation has been reported [119]. However, complete demethylation of the regulatory regions of the Foxp3 gene is required for stable Foxp3 expression linked to Treg cell function [120]. Traffic-related particles have been found to exert epigenetic modulation even when exposure occurs via the placenta [121].

Though early life is important for epigenetic modulation, such changes also occur in adults [122], which is not unexpected, given the inherent plasticity of the epigenetic mechanisms. This plasticity also provides for an opportunity for environmental/behavioural strategies in disease prevention [123].

Although the imbalance in regulatory *versus* inflammatory circuits is a universal feature in various inflammatory conditions, the basic mechanisms have been examined in greatest detail in allergic diseases, the focus of the following discussion.

### Tolerance – the key issue in allergy prevention

The concept of induction of immune tolerance has become a prime target for prevention and treatment strategies for many diseases such as allergy, asthma, autoimmunity, organ transplantation and infertility in which dysregulation of the immune system plays an essential role. In addition to various modes of allergen-SIT, healthy immune response development during high dose of allergen exposure in beekeepers and cat owners have been intensively studied to understand mechanisms of allergen tolerance in humans [124,125]. Mechanisms include changes in the profile of allergen-specific memory T and B cell responses, and specific antibody isotypes towards a non inflammatory direction, as well as decreased activation, tissue migration and degranulation of mast cells, basophils and eosinophils [10,126,127].

In different guidelines and consensus reports little attention is devoted to immunological tolerance and how to strengthen this in the prevention and management of allergic diseases. This is the case in spite of the fact that basic immunological mechanisms of tolerance have largely been unravelled and the commonly used strategy to reduce allergen exposure at different stages from the inception of an allergic disease to its life-long expression (allergen avoidance) is of questionable efficacy. With the possible exception of occupational exposures, effective allergen avoidance is difficult, if not impossible, and the results from the avoidance trials have been mostly discouraging [128,129]. Although there is some evidence that multiple allergen reduction strategies are more successful, at least for primary prevention [130], complex and multifaceted avoidance procedures cannot be seen as a long term solution for a common public health problem [131].

It has been generally thought that the dose-response curve for gaining immunological tolerance to different allergens/ bioparticles is different. However, the emerging picture is that a non-linear bell-shaped dose-response curve is a universal phenomenon, shown at least for endotoxin, fungal 1-3-beta-glucans, cat and dog allergens, rat and mouse allergens and house dust mite allergens (reviewed in [128]). In several recent reviews, the mechanisms in the development of tolerance have been comprehensively considered [132-134].

Clinical tolerance in its broadest terms comprises not only immunological tolerance, but also tolerance affecting tissues and cells other than the immune system, including psychological tolerance. Mild, intermittent allergy can be viewed as a trait or characteristic rather than a state requiring regular treatment or strict instructions for allergen avoidance. Mild allergy does not necessarily worsen with time [135], but often, particularly in children, wanes naturally through childhood and adolescence.

### Practical actions to promote allergy health

**Figure 3:**
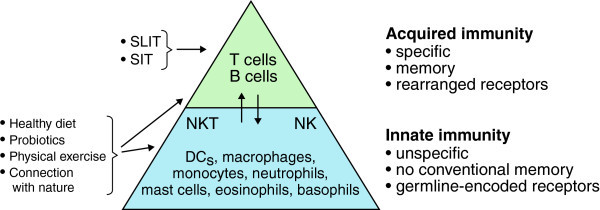
**Tolerance can be endorsed by behavioral means including physical training, consumption of healthy diet and doing activities in natural environments.** These things should be included also in secondary prevention together with specific immunotherapy. Unspecific and specific means to endorse tolerance act in synergy. Natural Killer (NK) cells and Natural Killer T (NKT) cells share characteristics of both innate and acquired immunity. SIT=Specific Immunotherapy, SLIT=Specific Oral Immunotherapy.

There are no clearly established guidelines for primary prevention of allergic disease or any established methods to non-specifically strengthen tolerance in established disease. However, data have accumulated to indicate that some simple behavioral activities can confer some protection against or alleviate allergic diseases, providing indirect evidence of their beneficial effects on tolerance. Not unexpectedly, such interventions include physical exercise, a healthy diet and connection with the natural world and countryside. Acting in synergy with specific immunotherapy, enhancing tolerance by these means is important also in secondary prevention (Figure 3).

In Western societies, chronic inflammatory diseases affect an increasing proportion of the population. In prevention and disease management, studies that highlight the immunological and anti-inflammatory effects of environmental- and life-style factors are urgently needed, particularly in view of this increased inflammatory burden (Tables 2, 3).

**Table 2:** **Practical advice to build-up and improve tolerance for primary prevention **[157]

**Primary prevention**	
• Support breastfeeding, solid foods from 4–6 months.	
• Do not avoid environmental exposure unnecessarily (e.g. foods, pets).	
• Strengthen immunity by increasing connection to natural environments.	
• Strengthen immunity by regular physical exercise.	
• Strengthen immunity by healthy diet, e.g. traditional Mediterranean or Baltic type.	
• Use antibiotics only for true need, majority of microbes are useful and build-healthy immune function.	
• Probiotic bacteria in fermented food or other preparations may strengthen immune function.	
• Do not smoke, e.g. parents smoking increase asthma risk in children.	

**Table 3:** **Practical advice to build-up and improve tolerance for symptom prevention (secondary prevention) and to prevent exacerbations/attacks (tertiary prevention)**[157]

**Secondary and tertiary prevention**	
• Regular physical exercise is anti-inflammatory	
• Healthy diet is anti-inflammatory, e.g. traditional Mediterranean or Baltic type of diet improves asthma control.	
• Probiotic bacteria in fermented food or other preparations may be anti-inflammatory.	
• Allergen specific immunotherapy:	
**-** allergens as is (foods)	
**-** sublingual tablets or drops (pollens, mites)	
**-** subcutaneous injections (e.g. insect stings)	
• Hit early and hit hard respiratory/skin inflammation with medication. Find treatment for long-term control.	
• Do not smoke, asthma and allergy drugs do not have full effects in smokers.	

#### Physical activity

Several outlines of evidence emphasize the risks of a sedentary lifestyle for health. Physical inactivity increases the inflammatory burden, independently of obesity, and, conversely, exercise programs decrease systemic low-level inflammation in various patient groups and healthy adults (reviewed in [136]). The anti-inflammatory effects of physical exercise seem to be mediated by activating the regulatory circuits, including regulatory T cells. For example, in healthy adults, a 12 week program of moderate exercise 3 times per week significantly improved regulatory T cell numbers and function [137]. Moderate exercise for 12 weeks in patients with type 2 diabetes, significantly decreased HbA1c levels while increasing IL-12 levels and the expression of transcription factors T-bet and Fox3p^+^, indicating improvements both in the metabolic and the immune systems [138]. In asthmatic children, a 12-week exercise program decreased total- and house dust mite specific IgE levels [139]. The anti-inflammatory effect of exercise has also been demonstrated in a murine model of asthma [140]. Physical activity is an especially important means for primary allergy prevention in children. In particular, children with asthma benefit from moderate physical activity, as they have frequently been found to be more inactive than their non-asthmatic peers [141].

#### Healthy diet

In addition to micro-organisms, dietary factors have been extensively studied to uncover possible additional factors behind the asthma and allergy epidemics in modern urban environments, given the modulatory potential of nutrients on epigenetics, intestinal microbiota and immune function [142]. While an inverse link between various nutrients or vitamins and occurrence of allergic diseases has been proposed in cross-sectional studies, the results are inconsistent and inconclusive. However, a systematic review based on 62 reports and 11 databases concludes that although the epidemiologic evidence thus far is weak, it supports a beneficial role for increased consumption of more fruits and vegetables, associated with better asthma and allergy outcomes. For example, a traditional Mediterranean diet confers protection against persistent wheeze and atopy [143]. It must be also born in mind that at least a part of the beneficial effect of fresh fruit and vegetables may be mediated by micro-organisms abundantly present on their surfaces [144].

Vitamin D deficiency has been suspected to play a role in the ´asthma epidemic´ as it may influence genomic programming of fetal development and subsequent disease risk and is tightly linked to diet and exposure to sunlight. This hypothesis is in the process of being tested in a large controlled trial in pregnant women and their offspring for the primary prevention of asthma [145,146].

Altogether, little is known of the mechanisms involved in the effects of healthy diets on the origins and progression of allergy or associated diseases. With respect to unhealthy food and overeating, preliminary data are available to show that obesity in mice and humans leads to Treg cell depletion in adipose tissues which, in turn, is associated with adipose tissue inflammation in obese individuals [147].

#### Probiotics

The benefits of probiotics to prevent or treat allergic diseases and asthma remain inconclusive. Bacteria-based products hold great promise for allergy prevention, but in the case of probiotics, the most beneficial bacterial strains, doses, duration and timing of supplementation are not determined [148-150]. A controlled clinical trial of about 1000 high allergy risk infants indicates that a probiotic mixture for 6 months has some protective effect against IgE associated (atopic) eczema at 2 years. This effect disappeared at 5 years, but remained in caesarean section -delivered children [151]. Probably a more or less continuous stimulation of innate immunity is needed to build up and maintain tolerance, not only intervention of 6 months.

#### Connection with nature and the natural environment

An urban environment appears to lack elements that apparently are important for the proper development of immune tolerance. The recognition of the (absolute) dependence of humans on both the commensal and environmental microbiota is crucial to unravel the mechanisms involved. In recent years, concepts such as ‘ecotherapy’ [152], ‘green exercise’ [24] and ‘forest therapy’ [27] have been launched. Urbanization and densification policy continues globally, and within the next 30 years, it is estimated that two-thirds of the world’s population and 85% of the population in the developed countries will live in urban areas with little green space [153]. Prevalence of inflammatory diseases is likely to increase even more. The health effects of nature and green spaces (see ‘Biodiversity and human health’) should be recognized, and measures to limit excessive land use and fragmentation urgently undertaken.

### Need for a global allergy plan

**Figure 4:**
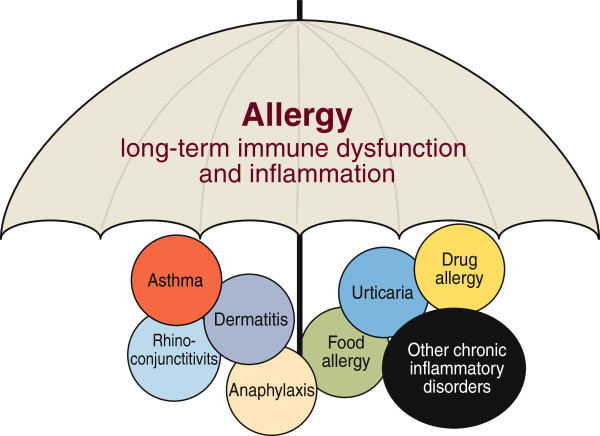
A variety of age-dependent and organ specific clinical allergic manifestations - which often occur together in the same predisposed individuals - contribute to the growing global allergy burden.

Success in several asthma programs in various countries have shown that a change for the better can be achieved with a pragmatic population-based action plan with set goals [154,155]. For allergy, including variable conditions from asthma to food allergy and anaphylaxis, one such comprehensive, nationwide plan has been successful [128,156,157]. In Finland, the allergy initiative collaborated with the government (both central and local) and several national and international organizations as well as non-governmental patient organizations to decrease the burden of all allergic conditions. Such an allergy action plan is a challenging task given the multifaceted and complex nature of the entity ‘allergy’ (Figure 4).

The rationale for such a program must stand on two equally important pillars - the best scientific evidence and a broad clinical experience. The goals and focus of the plan have to target the central problems, the plan and strategies need to be well defined and evaluation methods defined. In order to address this escalating major global health challenge, WAO has taken steps in this direction with the recently published WAO White Book on Allergy which provides a comprehensive view of the problem, includes reports from its national member societies about the current state of allergy/immunology resources in their countries, and offers recommendations for action [158].

### Concluding remarks

An ever-growing proportion of populations suffer from chronic inflammatory diseases, also called non-communicable diseases [159], as a result of mismatched immunological mechanisms and adaptation to modern urban life. Environmental micro-organisms, previously ubiquitous and abundantly present e.g. in our food, drinking water and milk, are key players for the induction and maintenance of immunoregulatory circuits and tolerance [11,50]. Our life-style and environment have a constant and continuous effect on the composition and dynamics of our microbial communities, and on the innate and adaptive immunity as the result. The total exposition matters: through gastrointestinal tract, vagina, skin and airways.

Two independent lines of research, i) metagenomic studies of the gut microbiota (and increasingly of other sites) and ii) immigrant studies, indicate that inflammatory diseases characteristic of modern life in affluent countries are associated with changes in the environmental and indigenous microbiota. Reduced diversity and changing composition of micro-organisms in environments is a consequence and a part of the more global problem of disappearing natural environments and general loss of biodiversity. The ´far out biodiversity´ (plant and animal life) and the ´close to biodiversity´ (microbiotas) are interconnected and shrinking.

**Figure 5:**
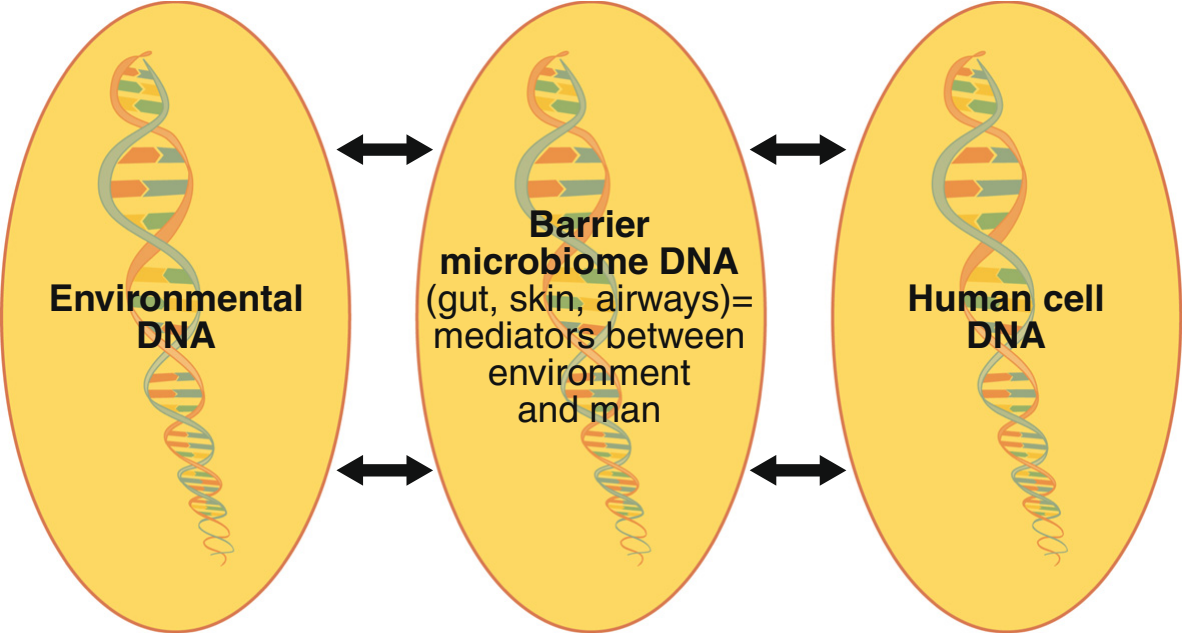
The interactions, “cross-talk” of the three cellular DNA compartments determines human survival.

Modern research focuses on microbiotas inhabiting the barriers of man and environment. The genetic composition of the barrier microbiotas, microbiomes mediate the signalling between human DNA and environmental DNA. It is this cross-talk, which we are only starting to explore, that determines our survival; the capability of the human immune system to make the difference between danger and non-danger, and the difference between self and non-self (Figure 5). The barrier microbiomes can be regarded as the “second genetic reservoir” of man, co-existing as a result of co-evolution of millions of years.

Pressure caused by the ever growing human populations has direct effects through habitat destruction and indirect effects through climate change [160]. Climate change has the potential to increase aeroallergens such as pollen and mold spores by earlier start of pollen season, increased allergenicity, and changes in pollen spatial distribution. These changes adversely impact allergic diseases.

Air pollution, both outdoors and indoors, is a severe global problem and needs full attention. It may break mucosal tolerance in several ways. However, even in cities with a relatively clean and controlled air, occurrence of allergies is high, and even higher than in cities with more air pollution problems. It is probable that poorly developed immunological tolerance is more of a basic factor on which many secondary factors, like air pollution, build their burden. The interactions are complex and poorly understood.

The key question is how to maintain and restore tolerance in various populations, which, along with globalization, are more movable than ever? Specific immunotherapy (SIT) was introduced 100 years ago [161] and has evolved in many ways. Restoration of tolerance is partly possible even in diseased individuals. But SIT may work even better, if the mechanisms of innate immunity are targeted more effectively. No established means for primary prevention are available, albeit bacteria-based products hold some promise.

The Global Allergy Plan could be a powerful tool to increase awareness of the global public health problem and combat the high burden of allergies. It may also have a preventive effect on other non-communicable diseases.

The biodiversity hypothesis can be regarded as an extension of hygiene [162,163] or “old friends” hypothesis [1,11] and microbial deprivation or microbiota hypothesis [164]. Population growth (urbanization) leads to loss of biodiversity (poor macrobiota/microbiota), poor human microbiota (dysbiosis), immune dysfunction (poor tolerance), inflammation and finally to clinical disease.

### Definition of terms

Biodiversity The variability among living organisms from all sources, including, *inter alia,* terrestrial, marine and other aquatic ecosystems and the ecological complexes of which they are part this includes diversity within species, between species and of ecosystems

Commensal A member of the normal flora

Dysbiosis Reduced diversity and disturbed composition of the microbial community within a given niche

Epigenetic changes Functionally relevant genome modifications, induced by environmental stimuli, that can be heritable, but are unrelated to DNA sequence changes

Indigenous Within the person (synonymous to ‘commensal’ flora)

Macrobiota The living organisms of a region that are large enough to be seen with the naked eye.

Metagenomic Refers to all of the genetic material of a microbial community sequenced together.

Microbiome All of the genetic material of micro-organisms within a given niche. Usually refers to bacteria but can include also genomes of fungi, viruses and protozoans

Microbiota The assemblage of micro-organisms within a given niche. Usually refers to bacteria, but can include also fungi, viruses and protozoans

Saprophyte A non-pathogenic environmental bacterium dependent on degrading plant material

### Note

Reference [165]; see the legend of Figure 1.

References [166-168]; see the legend of Figure 2.

## Competing interests

The authors declare that they have no competing interests.

## Authors’ contributions

TH led the development of the paper. All authors contributed evidence and analysis in their areas of expertise. WAO regional member organizations participated in the development and review of the manuscript: AAAAI, ACAAI, SLAAI, APAAACI, EAACI. All authors read and approved the final manuscript.
